# Modelling the host–pathogen interactions of macrophages and *Candida albicans* using Game Theory and dynamic optimization

**DOI:** 10.1098/rsif.2017.0095

**Published:** 2017-07-12

**Authors:** Sybille Dühring, Jan Ewald, Sebastian Germerodt, Christoph Kaleta, Thomas Dandekar, Stefan Schuster

**Affiliations:** 1 Department of Bioinformatics, Friedrich-Schiller-University Jena, Jena, Germany; 2 Research Group Medical Systems Biology, Institute for Experimental Medicine, Christian-Albrechts-University Kiel, Kiel, Germany; 3 Biocenter, Department of Bioinformatics, Julius-Maximilians-University Würzburg, Würzburg, Germany

**Keywords:** game theory, *Candida albicans*, dynamic optimization, non-lytic expulsion, macrophage replication, pathogenic fungi

## Abstract

The release of fungal cells following macrophage phagocytosis, called non-lytic expulsion, is reported for several fungal pathogens. On one hand, non-lytic expulsion may benefit the fungus in escaping the microbicidal environment of the phagosome. On the other hand, the macrophage could profit in terms of avoiding its own lysis and being able to undergo proliferation. To analyse the causes of non-lytic expulsion and the relevance of macrophage proliferation in the macrophage–*Candida albicans* interaction, we employ Evolutionary Game Theory and dynamic optimization in a sequential manner. We establish a game-theoretical model describing the different strategies of the two players after phagocytosis. Depending on the parameter values, we find four different Nash equilibria and determine the influence of the systems state of the host upon the game. As our Nash equilibria are a direct consequence of the model parameterization, we can depict several biological scenarios. A parameter region, where the host response is robust against the fungal infection, is determined. We further apply dynamic optimization to analyse whether macrophage mitosis is relevant in the host–pathogen interaction of macrophages and *C. albicans*. For this, we study the population dynamics of the macrophage–*C. albicans* interactions and the corresponding optimal controls for the macrophages, indicating the best macrophage strategy of switching from proliferation to attacking fungal cells.

## 1. Introduction

*Candida albicans* is one of the most frequent opportunistic pathogens of humans, and can infect many body sites [1–3]. In response to environmental challenges, *C. albicans* can change its morphology from yeast to hyphal growth and back [3–5]. Hyphae formation and the invasion of host tissue are seen as determinants of the shift from a commensal to an invasive pathogen [6]. In immunocompetent hosts, the defence against *C. albicans* mainly relies on the innate immune system, especially neutrophils and macrophages [7–9]. *Candida albicans*, on the other hand, has developed a plethora of response and evasion mechanisms to circumvent recognition by the immune system and to control, evade and interfere with the host immune reactions [3,7]. An overview of the immunological cross-talk between *C. albicans* and the innate immune system can be found in Dühring *et al.* [10]. In this article, we focus on the interaction of *C. albicans* and macrophages.

Macrophages are dynamic immune cells that are disseminated in several tissues of the human host [11]. The number of macrophages at an infection site is mainly influenced by invading monocytes that differentiate into macrophages. The proliferation of mature macrophages is rather slow and rare but is observed repeatedly for different types of macrophages, e.g. human alveolar macrophages [12–14], murine hepatic macrophages (Kupffer cells) [15,16], murine peritoneal macrophages [17,18] and the J774.1 macrophage cell line [19]. It is also of importance in chronic inflammation [13,14,18]. Being a special type of phagocyte, macrophages can phagocytose and eliminate the round yeast cells and relatively short hyphae of *C. albicans* and thereby limit the fungal burden early upon infection [8,11,20]. Inside the macrophage phagosome, some *C. albicans* cells are killed through oxidative and non-oxidative microbicidal mechanisms such as by diminution of the pH [2,8,11]. However, *C. albicans* hyphae and yeast cells can manipulate the phagosomes from the inside by raising the pH [9,21]. This leads to germination of *C. albicans* yeast cells and hyphae formation within the macrophage [7,9,22]. While the *C. albicans* yeast cells fail to damage the macrophage, the elongating *C. albicans* filaments can lead to macrophage death by lysis, allowing the fungus to escape [5,22,23]. Hence, macrophages and *C. albicans* can have a significant cytotoxic effect on each other [8]. Yet, the response of *C. albicans* to the phagocytosis by macrophages depends on the genomic background of the fungus. Distinct karyotypes (b and c) of *C. albicans* isolates differ in their ability to resist intracellular killing. The c karyotype isolates are reported to be more resistant to intracellular killing and to escape from within macrophages by killing the immune cell. This c karyotype is thus considered to behave in a more virulent (or aggressive) way than the b karyotype [2,24,25].

There also exists an alternative escape of hyphae-producing *C. albicans* cells from within macrophages, called non-lytic expulsion (also termed exocytosis, extrusion or vomocytosis). Shortly after a phagocytosed *C. albicans* cell starts to form hyphae, it is released into the medium by the macrophage. After non-lytic expulsion both *C. albicans* and the macrophage are capable of surviving. The host macrophage and the expelled *C. albicans* cell appear morphologically normal. The macrophage continues to undergo mitosis and the *C. albicans* hypha extends at normal rates [26–29]. Non-lytic expulsion of fungal cells following phagocytosis by macrophages was first described in *Cryptococcus neoformans* [29–31]. This process is also observed in *Candida krusei* [29,32,33]. Even though the frequency of non-lytic expulsion can be low (in *C. albicans* less than 1%), the event is observed under various experimental conditions [26,31,34,35]. Furthermore, Nicola *et al.* [34] showed in *C. neoformans* that non-lytic expulsion is not an *in vitro* artefact, but occurred *in vivo* in murine models with a frequency that is assumed to be much higher than that observed *in vitro* [29,33,34].

This gives rise to several questions: under which conditions is non-lytic expulsion beneficial to the macrophage and/or the fungus? On one hand, the pathogen is able to escape the hostile environment of the macrophage phagosome. On the other hand, the macrophage is able to avoid potential lysis. Another potential advantage of non-lytic expulsion to the macrophage is the possibility of undergoing mitosis, which can fail in macrophages with phagocytosed *C. albicans* cells [19,26]. This in turn leads to the question of under which conditions and to what proportions of the macrophage population is it beneficial to the macrophages to undergo mitosis? And further, is it better for macrophages to try to phagocytose and kill *C. albicans* cells straight away or to proliferate first and switch to phagocytosis at a later time point, when (locally) present in larger numbers? Both dynamic regimes have their advantages. Phagocytosing straight away reduces the number of *C. albicans* cells earlier upon infection. Proliferating first increases the number of macrophages, which provides an advantage later. To analyse the causes of non-lytic expulsion and the relevance of macrophage proliferation in the macrophage–*C. albicans* interaction, we employ two complementary approaches of mathematical modelling in a sequential manner: Evolutionary Game Theory and dynamic optimization.

Optimality principles are often used to study and explain biological processes [36,37]. Originating in engineering, dynamic optimization has been used successfully to find optimal regimes in several biological systems including infection processes [38–42], protein assembly [43], metabolic pathways [44] and to optimize medical applications such as the treatment of cancer [45] or diabetes [46]. In the theoretical description and modelling of host–parasite interactions, Evolutionary Game Theory has turned out to be a very useful tool [47–51]. Game Theory is used, in particular, to describe diverse *C. albicans* interactions [49,52,53]. Hummert *et al*. [49] studied the optimal survival strategy of *C. albicans* cells after phagocytosis by a macrophage. In their setting, *C. albicans* cells play against each other, while the macrophage is considered as a constant environment. Tyc *et al*. [52] analysed the coexistence of yeast and hyphal forms in a *C. albicans* population. In a later study by Tyc *et al*. [53], the colonization dynamics of *C. albicans* cells expressing different levels of *EFG1* in response to the host immune status are presented. In contrast with these previous works, we consider the host (more specifically, macrophages) as an active player and not solely as an environment.

In §2.1, we establish a game-theoretical model describing the different strategies of macrophages and *C. albicans* after phagocytosis. Depending on the parameter values, we determine the Nash equilibria (solutions of the game) and analyse the influence of the systems state of the host upon the game (see §3.1). As our Nash equilibria are a direct consequence of the model parameterization, we can depict several biological scenarios. We further determine a parameter region, where the host response is robust against this fungal infection. In §2.2, we apply dynamic optimization to analyse whether macrophage mitosis is relevant in the host–pathogen interaction of macrophages and *C. albicans*. In this way, we attempt to clarify what regime (phagocytosing or proliferating first) the macrophage population should apply. For this, we determine the population dynamics and corresponding optimal controls indicating the best macrophage strategy of switching from proliferation to attacking fungal cells by macrophages in §3.2.

## 2. Material and methods

### 2.1. Characterization of the game

In Evolutionary Game Theory, both the host and pathogen are considered as evolutionary antagonistic players which can show different strategies to maximize their fitness. These strategies can be, for example, cellular traits like the up- and down-regulation of metabolic pathways, expression of virulence factors or generation of different splice variants [50]. A change in strategy can occur by mutation, epigenetic modifications, stochastic gene expression or due to the immunological cross-talk of the players. Each player's fitness is quantified as the net payoff of costs and benefits. Although it is often difficult to quantify the payoff, it is taken into account that the payoff for each player not only depends on its own strategy but also on that of the antagonistic counterpart(s). The solutions of the game are called Nash equilibria. Intuitively, the classical and frequently used concept of the Nash equilibrium is a situation in which neither of the players has an incentive to change strategy unilaterally [47,54].

Game Theory can even be applied if no changes between strategies occur, and should then be interpreted in view of population games. A population may consist of subpopulations determined, for example, by different alleles or karyotypes. These subpopulations can be characterized by different strategies. The final strategy observed in the pure Nash equilibrium arises because one subpopulation outcompetes the other. In the case of a mixed Nash equilibrium, all subpopulations coexist in oscillatory or stationary ways.

**Figure 1.**
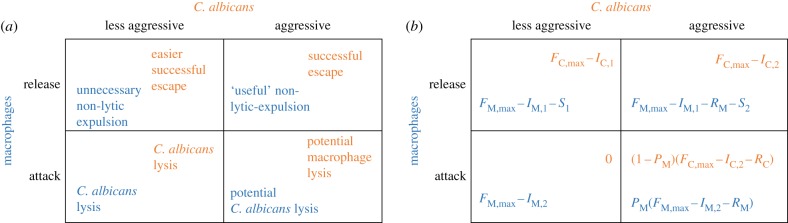
Characterization of the game after phagocytosis: each player's strategies and possible outcomes (*a*); the payoff functions in terms of fitness *F* for the *C. albicans* cell (orange) and the macrophage (blue) (*b*).

In this study, we followed the game-theoretical framework proposed by Renaud & De Meeüs [48] and adapted it to the specific situation after a macrophage (player I) has phagocytosed a *C. albicans* cell (player II). During this confrontation, each of the two players has two strategies (figure 1*a*). The macrophage can either ‘release’ the *C. albicans* cell (non-lytic expulsion) or ‘attack’ and try to kill the pathogen.

The *C. albicans* cell can either be ‘less aggressive’ (e.g. karyotype b as described in §1) or ‘aggressive’ (e.g. karyotype c as described in §1). Hereafter, we assume that a macrophage is always able to win a confrontation with a ‘less aggressive’ *C. albicans* cell. This is not necessarily the case when a macrophage plays against an ‘aggressive’ *C. albicans* cell.

Pairing the different strategies leads to four distinct types of confrontation: ‘release’/‘less aggressive’, ‘release’/‘aggressive’, ‘attack’/‘less aggressive’ and ‘attack’/‘aggressive’. Each player's payoff for the different confrontations is recorded in terms of fitness *F* of individuals (figure 1*b*). The maximum fitness an individual can obtain in a population is denoted by *F*_max_. The macrophage's payoffs are referred to as *F*_M_, while the *C. albicans'* payoffs are referred to as *F*_C_. For our considered confrontations this gives the following pattern.

‘attack’/‘aggressive’: The payoff of the macrophage is2.1
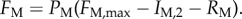
The payoff of the *C. albicans* cell is2.2
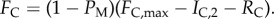
Only one of the two players can survive in this setting. The probability that the macrophage wins the confrontation is denoted by *P*_M_. Hence 0 < *P*_M_ < 1, with (1 − *P*_M_) being the probability that the fungus wins the confrontation. Both players have investment costs to play their strategy, denoted by *I*_M_ and *I*_C_. These could be (but are not limited to) the costs for morphological adaptation, e.g. hyphae development. Each player also has to invest in resisting its opponent's attack. These resistance costs are given by *R*_M_ and *R*_C_.

‘attack’/‘less aggressive’: The macrophage's payoff is2.3
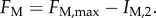
In this confrontation, the fungus is eliminated. Its payoff is therefore *F*_C_ = 0.

‘release’/‘aggressive’: Here, the payoff of the macrophage is2.4
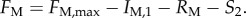
The payoff of *C. albicans* is2.5
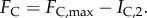
‘release’/‘less aggressive’: In this confrontation, the payoff of the macrophage is2.6
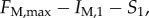
while the payoff of the pathogen is2.7
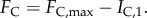


As the ‘aggressive’ *C. albicans* cell is much more virulent and resistant to the attacks of the macrophages, we assume that the investment costs *I*_C,2_ are higher than the investment costs *I*_C,1_. Hence, *I*_C,1_ < *I*_C,2_.

For macrophages to release a *C. albicans* cell can be more or less severe depending on the constitution of the host and the severity of the fungal attack. We therefore assume that the fitness consequences of the macrophage for ‘releasing’ a *C. albicans* cell are directly linked to the overall conditions of the host. This is implemented by the systemic costs *S*_1_ (‘releasing’ a ‘less aggressive’ fungal cell) and *S*_2_ (‘releasing’ an ‘aggressive’ fungal cell). These systemic costs represent the costs of the host (and not just the macrophage). This enables us to investigate the outcome of the game (i.e. the location of the Nash equilibria) in the light of the overall host conditions. Taking into account the overall systemic consequences also allows us to investigate the game-theoretical conflicts between the cell level and organism level, e.g. the seemingly erroneous expulsion of an ‘aggressive’ *C. albicans* cell by a macrophage to escape lysis. We assume the systemic costs *S*_1_ and *S*_2_ to be low when the host benefits from ‘releasing’ the fungus. Lewis *et* *al*. [19] postulated that non-lytic expulsion might enable macrophage mitosis to proceed normally. As macrophage replication can fail while a *C. albicans* cell is ingested inside a macrophage, the proliferating macrophage has an incentive to release the fungal cell. Hence, in a scenario where macrophage proliferation is beneficial to the host the systemic costs *S*_1_ and *S*_2_ are assumed to be low.

The resulting Nash equilibria of our game are given in §3.1. In §2.2 and §3.2, we investigate under which conditions proliferation is relevant for the macrophage–*C. albicans* interaction.

### 2.2. The dynamic optimization model

**Figure 2.**
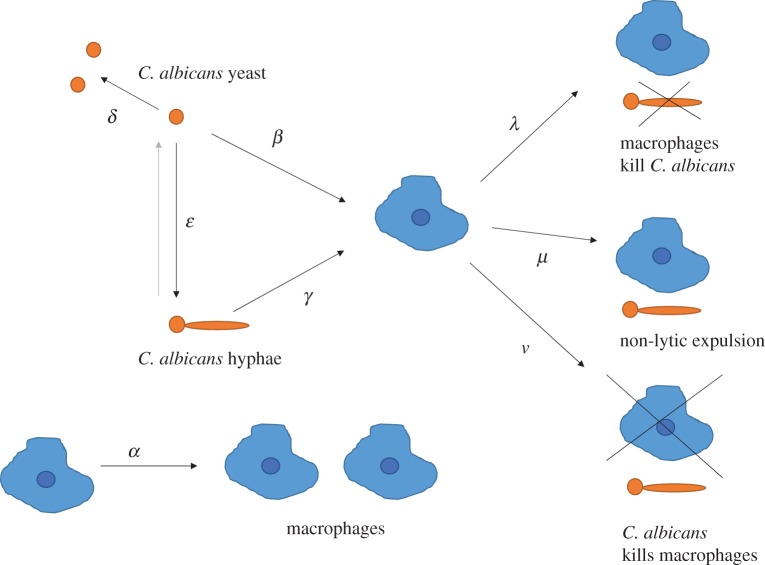
*Candida albicans* versus mammalian macrophages and the parameters of the differential equation model. *C. albicans* (orange) can proliferate and switch between its yeast and hyphal forms. Macrophages (blue) can undergo mitosis or phagocytose the fungus. There are three different outcomes after phagocytosis: the fungal cell is dead, both cells are alive and separated (non-lytic expulsion), or the macrophage is dead. The backward switch of *C. albicans* from hyphae to yeast (grey) is not represented in the differential equation model, as we are considering the pathogenic state of the fungus.

In contrast with Evolutionary Game Theory, dynamic optimization tries to identify a time optimal control in biological systems. To predict an optimal regime of phagocytosis and proliferation, we started by setting up a first-order ordinary differential equation model to simulate the complex dynamics of the host–pathogen interactions (figure 2). We further defined constraints and an objective function to perform dynamic optimization.

As macrophages are unable to replicate while they are phagocytosing, we are interested in an optimal strategy of the macrophage population with respect to mitosis versus phagocytosis. Hence, we are looking for a control *u*(*t*) ∈ [0, 1] that describes the optimal proportion of the macrophage population undergoing phagocytosis at a given point in time *t*.

In our model, the macrophage population is denoted by *m*. The symbol *y* stands for the *C. albicans* yeast cell population, *h* for the *C. albicans* hyphal cell population and *p* for the phagocytosed *C. albicans* cell population. For all populations, our model considers countable cells instead of biomass. In this way, we can use experimental data from the literature, where relevant rates are given as cells per hour (table 1). The differential equation system describing the considered macrophage–*C. albicans* interactions is formulated as follows:2.8
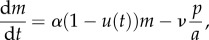
2.9
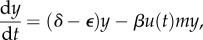
2.10
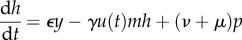
2.11
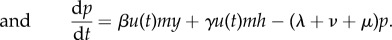
The proportion of proliferating macrophages is 1 − *u*(*t*). The macrophage proliferation rate is given by *α* (table 1). We assume that the number of fungal cells a macrophage is able to ingest is limited to *a* (table 1). Phagocytosed *C. albicans* cells kill macrophages at a rate *ν* and are killed at a rate *λ*. They are released via non-lytic expulsion at a rate *μ*. As a consequence, *ν*(*p*/*a*) macrophages are killed in each time step. The yeast cells proliferate at a rate *δ* and switch to the hyphal growth form at a rate *ε*. They are phagocytosed by macrophages at a rate *β*. *Candida albicans* hyphal cells are phagocytosed at rate *γ*. We here assume that all phagocytosed *C. albicans* cells form hyphae inside macrophages so that there are no cells remaining in yeast form. Those *C. albicans* cells that escaped from the phagosome by killing the macrophage are therefore added to the hyphae population. As a reasonable reduction in complexity, our model does not consider hyphal elongation. However, the qualitative behaviour of our results should not be affected by this, as macrophages are only able to phagocytose relatively short hyphae.

**Table 1.** Summary of derived mean values for the parameter distribution of our optimization problem.

parameter	description	value	source
*α*	macrophage replication rate per hour	0.0176 (standard, average value without the outlier), 0.059 (high, average value with the outlier) and 0.266 (maximal, the outlier)	[12–14,16,18,19]
*β*	yeast-phagocytosing rate by macrophages per cell and hour	0.675	[9,55,56]
*γ*	hyphae-phagocytosing rate by macrophages per cell and hour	assumed to be 0.431	following [57]
*δ*	yeast replication rate per hour	0.0393	[58–60]
*ε*	filamentation rate per hour	0.2705	[5,9,61,62]
*μ*	non-lytic expulsion rate per hour	0.00166	[26]
*ν*	macrophage-killing rate by *C. albicans* per hour	0.0676	[5,9,62–64]
*λ*	*C. albicans*-killing rate by macrophages per hour	0.0797	[9,56,65–69]
*a*	number of *C. albicans* cells inside one macrophage at a time	3.28	[5,56,63,65,70]

As the fungal burden should be minimal at all times, we minimize the integral of all *C. albicans* cells outside macrophages2.12
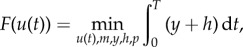
subject to the differential equation system (equations (2.8)–(2.11)) and the following inequality constraints:2.13
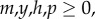
2.14
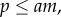
2.15
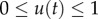
2.16
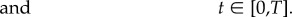


## 3. Results

### 3.1. Solution of the game

In this section, we determine the Nash equilibria for our game of the interaction between a macrophage (player I) and a *C. albicans* cell (player II).

The payoff matrix for the macrophage is3.1
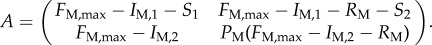


The payoff matrix for the fungus is3.2
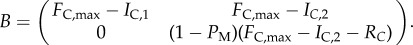


If the macrophage plays its ‘attack’ strategy, the *C. albicans* cell has to adopt the ‘aggressive’ strategy, whatever the average expected value of (1 − *P*_M_)(*F*_C,max_ − *I*_C,2_ − *R*_C_) (equation (2.2)), as the fungus is otherwise eliminated. If the macrophage plays its ‘release’ strategy instead, the fungus will play its ‘less aggressive’ strategy as *I*_C,1_ < *I*_C,2_.

Depending on the values of the parameters *I*_M,1_, *I*_M,2_, *S*_1_, *S*_2_ and the probability *P*_M_ of winning against the fungus the macrophage chooses either ‘attack’ or ‘release’.

There are four different cases of solutions to the game (table 2). In case 1 and case 3, we find the strategy pair ‘attack’/‘aggressive’ as a pure Nash equilibrium, whereas in case 2 and case 3 non-lytic expulsion occurs as a pure Nash equilibrium. In case 4, we find a mixed Nash equilibrium, where each strategy is played with a certain probability so that ‘attacking’/‘releasing’ and ‘aggressive’/‘less aggressive’ cells on both sides coexist with certain frequencies in the population.

**Table 2.** Solutions of the game.

case	conditions	Nash equilibria
case 1 (figure 3*a*)	*I*_M,1_ + *S*_1_ > *I*_M,2_ and the average expected value of equation (2.1) is bigger than equation (2.4)	‘attack’/‘aggressive’
case 2 (figure 3*b*)	*I*_M,1_ + *S*_1_ < *I*_M,2_ and the average expected value of equation (2.1) is less than equation (2.4)	‘release’/‘less aggressive’
case 3 (figure 3*c*)	*I*_M,1_ + *S*_1_ < *I*_M,2_ and the average expected value of equation (2.1) is bigger than equation (2.4)	‘attack’/‘aggressive’ and ‘release’/‘less aggressive’
case 4 (figure 3*d*)	*I*_M,1_ + *S*_1_ > *I*_M,2_ but the average expected value of equation (2.1) is less than equation (2.4)	mixed Nash equilibrium

**Figure 3.**
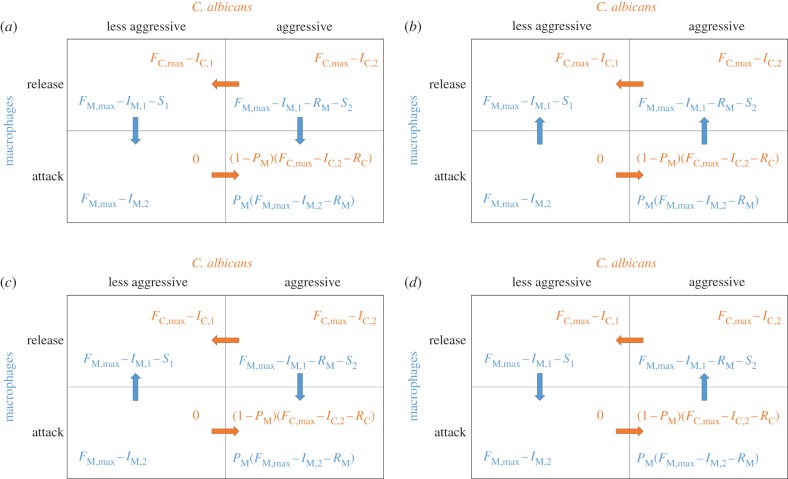
Nash equilibria of the game. Depicted are the four described cases with their corresponding Nash equilibria: ‘attack’/‘aggressive’ (*a*), ‘release’/‘less aggressive’ (*b*), ‘attack’/‘aggressive’ and ‘release’/‘less aggressive’ (*c*), mixed Nash equilibrium (*d*). Again, the payoff functions of the *C. albicans* cell are orange, while the payoff functions of the macrophage are blue. The arrows indicate the relations of the functions, pointing from the smaller payoff to the bigger payoff.

Referring to the payoff matrix *A* and *B* in equation (3.1) and equation (3.2), we can determine the mean expected payoff *E*_M_ for the macrophage as a player and *E*_C_ for the *C. albicans* player. The mean expected payoff for *C. albicans* is3.3
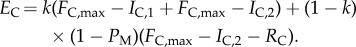


**Figure 4.**
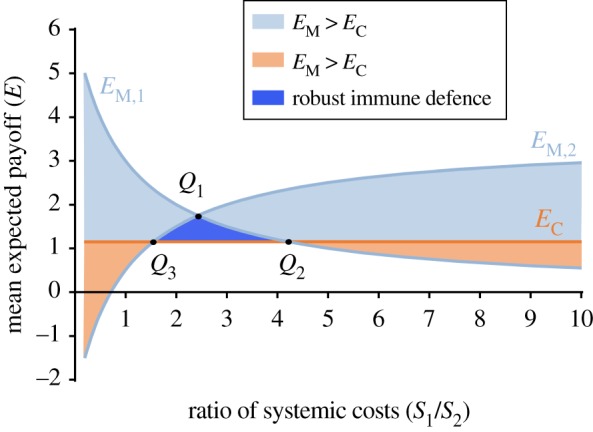
The mean expected payoff for *C. albicans* *E*_C_ is independent of the systemic costs of the host (orange line). The mean expected payoff of macrophages *E*_M_ depends on the temptation of *C. albicans* cells to play the ‘less aggressive’ strategy (*E*_M,1_) or the ‘aggressive’ strategy (*E*_M,2_). Depending on the deflection of the system state, represented by the ratio of the systemic costs *S*_1_/*S*_2_, *E*_M_ may be superior to *E*_C_ (blue area) or not (orange area). As long as the value of the mean expected payoff of the macrophage in point *Q*_1_ is bigger than the value of *E*_C_, a subspace of solutions, framed by *Q*_1_, *Q*_2_ and *Q*_3_, can be determined where the immune defence is considered to be robust and independent of the strategy of *C. albicans* cells.

The mean expected payoff *E*_C_ depends on the macrophage's tendency to ‘release’ (*k*) or to ‘attack’ (1 − *k*) the *C. albicans* cell. It is, however, independent of the systemic costs of the host (figure 4). The mean expected payoff for the macrophage is3.4
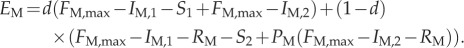
Here, *d* stands for the probability that an interacting *C. albicans* cell plays the ‘less aggressive’ strategy, whereas (1 − *d*) stands for the probability of playing the ‘aggressive’ strategy. The systemic costs *S*_1_ and *S*_2_ exclusively affect *E*_M_ but not *E*_C_.

For *E*_M_ > *E*_C_, the mean expected payoff of a macrophage is superior to that of a *C. albicans* cell (figure 4). If *C. albicans* cells exclusively play the ‘less aggressive’ strategy (*d* = 1), *E*_M,1_ falls monotonically in *S*_1_/*S*_2_. However, if interacting *C. albicans* cells exclusively play the ‘aggressive’ strategy, *E*_M_ > *E*_C_ is only true if *S*_1_≫*S*_2_ (see *E*_M,2_ in figure 4). Let *Q*_1_ be defined as the point of intersection of *E*_M,1_ and *E*_M,2_. Further, let *Q*_2_ be the point of intersection of *E*_M,1_ with *E*_C_ and *Q*_3_ be the point of intersection of *E*_M,2_ with *E*_C_. As long as the value of the mean expected payoff of the macrophage in point *Q*_1_ is bigger than the value of *E*_C_ a parameter region can be determined, where the immune response is robust and independent of *C. albicans*’ temptation to play the ‘aggressive’ strategy (0 ≤ *d* ≤ 1). Simultaneously, *Q*_2_ and *Q*_3_ indicate the maximum deflection of the host's state, where the immune defence can be considered as robust. Since *k* determines the value of *E*_C_, a maximization of the area of robust solutions can be achieved if the macrophage is preferably playing the (pure) ‘attack’ strategy.

### 3.2. Solution of the dynamic optimization problem

Our optimization problem of the interaction of macrophages and *C. albicans* in §2.2 consists of a time-dependent, continuous control *u*(*t*) and state variables (cell populations). To solve this dynamic optimization problem we used a quasi-sequential approach with an extension to handle approximation errors and moving finite elements [71], as in previous works [43,72]. In this gradient-based approach the optimization is repeated 100 times for each parameter set with random initializations of the control variable to avoid ending in local optima.

To determine physiologically relevant model parameters, we used experimental data taken from the literature (table 1). Based on these parameter values, we determined the optimal replication strategy for macrophages with varying multiplicity of infection (MOI) and macrophage replication rates *α*.

**Figure 5.**
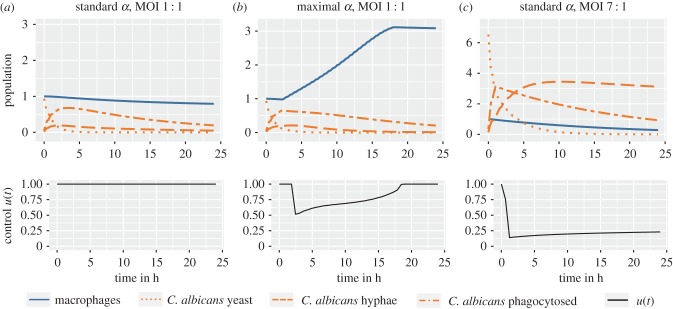
Dynamics of the population sizes of *C. albicans* cells and macrophages with standard and maximal macrophage replication rates and the corresponding optimal controls for different MOIs.

Our initial scenario assumes a standard macrophage replication rate *α* = 0.0176 and an MOI of 1 : 1 (*C. albicans* cells : macrophages). For this parameter setting, the optimal strategy of macrophages involves only phagocytosis and no replication (see control *u*(*t*) in figure 5*a*). The population dynamics reveal that macrophages are sufficient to control the number of *C. albicans* cells. During a simulated period of 24 h, the macrophage population drops only a little due to lysis. Since the replication rate of macrophages is rather low (*α* = 0.01757) in this setting, we repeated the optimization for the maximal macrophage replication rate reported in the literature (*α* = 0.2660; figure 5*b*). This scenario leads to a different optimal strategy *u*(*t*) starting with a phase of exclusive phagocytosis. This is followed by a phase of replication and phagocytosis and ends with a phase of exclusive phagocytosis. Comparing both scenarios, the number of *C. albicans* cells over the whole simulated time span is nearly identical. This indicates that, for the macrophages, replication (even with high rates) is of small advantage when the MOI is balanced. To see whether the amount of *C. albicans* cells rather than the replication rate influences the macrophages' strategy, we changed the MOI to 7 : 1, assuming again a standard replication rate (*α* = 0.0176; figure 5*c*). The resulting dynamic shows that macrophages are not able to control the invading *C. albicans* cells. The optimal strategy of macrophages switches immediately from exclusive phagocytosis to almost complete replication to escape lysis by *C. albicans*.

To quantify the influence of parameters on the optimal strategy of macrophages to combat *C. albicans* cells, we performed a parameter sensitivity analysis with varying MOIs (1 : 1 and 3 : 1) and macrophage replication rates (standard and high). As expected, the replication rate *α* has the highest impact on the amount of replication for the tested MOIs of 1 : 1 and 3 : 1 and replication rates *α*. This is followed by the *C. albicans* filamentation rate, *ε*, as well as the macrophage lysis rate by *C. albicans*, *ν* (electronic supplementary material, figures S1 and S2).

The corresponding optimal controls *u*(*t*) show for almost every parameter set no replication under the scenario MOI 1 : 1, where macrophages can efficiently control the *C. albicans* population (electronic supplementary material, figure S3). For the higher MOI of 3:1, more parameter sets lead to optimal strategies including phases of replication (electronic supplementary material, figure S4). This is because *C. albicans* cells exceed the manageable amount for macrophages in many parameter sets (electronic supplementary material, figures S1 and S2).

A closer inspection of both scenarios (MOI of 1 : 1, 3 : 1) reveals that replication is only effective if the replication rate exceeds a certain magnitude (electronic supplementary material, figures S1 and S2). The majority of optimal replication strategies have a similar time course to that seen in figure 5*b*. Therefore, we can conclude that an early phase of replication during the incubation of macrophages and *C. albicans* cells is only of advantage to the macrophage if the replication rate is very high. For more results on the sensitivity analysis see the electronic supplementary material.

## 4. Discussion

In this work, we used mathematical modelling to study non-lytic expulsion of *C. albicans* from within macrophages and analysed the relevance of macrophage replication in the macrophage–*C. albicans* interactions.

Applying dynamic optimization, we studied the population dynamics of the macrophage–*C. albicans* interactions and the corresponding optimal controls for the macrophages. To examine the conditions under which macrophage mitosis is beneficial to the macrophage population (and the host), we explored the dynamic regimes of phagocytosis and proliferation of macrophages in the light of an optimal response to fungal burden. For this, we looked into different infection scenarios represented by different MOIs (*C. albicans* : macrophages) and macrophage replication rates (see §2.2 and §3.2). Hereby, we considered cells instead of biomass for our populations.

Comparing the different scenarios, we found that replication (even with high rates) is of small advantage when the MOI is balanced (1 : 1). Our simulations indicate that, when macrophages are able to control the *C. albicans* invasion (balanced MOI of 1 : 1), the optimal strategy for macrophages includes almost no replication (figure 5*a*,*b*). Even with higher MOIs (3 : 1), replication is only effective if the replication rate exceeds a certain magnitude (electronic supplementary material, figures S2 and S4). When applying an MOI of 7 : 1, macrophages are no longer able to control the invading *C. albicans* cells regardless of the proliferation policy. In a healthy host with a standard macrophage replication rate (table 1), the great majority of macrophages solely phagocytose the fungal cells. Only with high macrophage replication rates, as, for example, seen in chronic inflammation, does the system's behaviour change and macrophage replication occurs in addition to phagocytosis.

We conclude that an early phase of macrophage replication during the incubation of macrophages and *C. albicans* cells is only of advantage to the macrophage if the replication rate is very high. In healthy individuals, macrophage replication is therefore of minor importance in this interaction.

We further analysed the conditions which render non-lytic expulsion beneficial for a given player (the macrophage and the fungus). For the fungus, non-lytic expulsion is always beneficial as the fungus escapes the hostile environment of the macrophage. For the macrophage, the situation is more nuanced. Using Evolutionary Game Theory, we found four different Nash equilibria depending on the investment costs of the macrophage, the systemic costs of the host and the probability that a macrophage is able to kill the fungus. From our game-theoretical model, we derived that non-lytic expulsion can occur as a pure Nash equilibrium (see cases 2 and 3 in table 2) as well as a mixed Nash equilibrium (see case 4 in table 2).

As our Nash equilibria are a direct consequence of the model parameterization, we can depict several biological scenarios. By adjusting the value of the systemic costs *S*_1_ for ‘releasing’ a ‘less aggressive’ fungal cell, it is possible to make a distinction between host conditions (i.e. healthy or weakened). In a healthy host, macrophage replication is of minor importance, as shown in our population dynamics in §3.2. Hence, the systemic costs *S*_1_ are high as the focus is on attacking the *C. albicans* cells and fungal clearance. The systemic costs *S*_2_ for ‘releasing’ an ‘aggressive’ fungal cell, on the other hand, are directly linked to the probability *P*_M_ of the macrophage winning the confrontation with the fungus. With decreasing chances of the macrophage surviving a fungal ‘attack’, the systemic costs *S*_2_ also decrease. The systemic costs *S*_2_ can thus function as a measure of the severity of the fungal attack. Note that there can exist a lag phase in the linkage of *P*_M_ and *S*_2_. During the advance of the *C. albicans* infection, the fungal cells become more difficult to handle for the macrophage because of hyphae formation. This can lead to a conflict between the host's cell and the organism level, especially when the macrophage's probability of losing to the fungus is increasing but the systemic costs *S*_2_ are still high. Our model predicts that, in this setting, the macrophage would still try to attack the fungus despite its decreasing chance of surviving such an attack. Only when the systemic costs *S*_2_ decrease to a low value does the strategy of the macrophage switch to non-lytic expulsion (see cases 2 and 4 in table 2) to avoid macrophage lysis. This loss in macrophage competence has to be compensated by other immune cells such as neutrophils (not part of the model presented here).

The scenario of an immune-competent host with a moderate fungal infection is best described by high systemic costs *S*_1_ while at the same time the systemic costs *S*_2_ and/or the probability *P*_M_ of winning against the fungus are high. In this parameter setting, we find the pure Nash equilibrium ‘attack’/‘aggressive’ (see case 1 in table 2). The macrophages’ focus is on attacking the fungal cells. A deviation from this strategy is blocked by the prohibitively high systemic costs of this scenario. ‘Attacking’ the fungus also maximizes the robustness of the immune response to *C. albicans*, as shown in §3.1. This leads to the immune state becoming more tolerant of perturbations by *C. albicans*.

The model also provides a scenario with a healthy host suffering from an advanced infection (severe fungal attack). The systemic costs *S*_1_ are still high but either the systemic costs *S*_2_ and/or the chance of ‘winning’ an attack against the *C. albicans* player are low (see case 4 in table 2). In this scenario, we find the mixed Nash equilibrium, which includes the release of fungal cells. It is noteworthy that only in this model scenario is an ‘aggressive’ fungal cell released instead of being killed.

In a weakened host (low *S*_1_), independent of the severity of the fungal infection (*S*_2_), we find non-lytic expulsion as a pure Nash equilibrium (see cases 2 and 3 in table 2). This is a direct consequence of the macrophage's costs for ‘attacking’ a ‘less aggressive’ fungus (*I*_M,1_ and *S*_1_) being higher than the costs for ‘releasing’ this type of fungus (*I*_M,2_) (see cases 2 and 3 in table 2).

From this we conclude that, for non-lytic expulsion to be beneficial to the macrophage, either the fungal ‘attack’ must be severe with a low probability of the macrophages surviving (as in the case of the mixed Nash equilibrium) or the host needs to have a strong incentive to release the fungus, i.e. to undergo mitosis. But, as mitosis is of minor importance to the macrophage–*C. albicans* interaction in a healthy host, the host conditions need to be in a state where the macrophage replication rate is strongly elevated, e.g. by chronic inflammation or by using a fast replicating macrophage cell line such as J774.1. To simulate a severe fungal ‘attack’ in experiments, it is not sufficient to only have a high fungal burden but rather to use an aggressive karyotype of *C. albicans*. As our simulations indicate, macrophages are not able to control the fungal burden with MOIs of 7 : 1. Instead, one would expect to see fungal outgrowth with a high occurrence of macrophage lysis. It would be of interest to further study the situation of chronic inflammation and/or severe fungal infection in wet lab experiments, as non-lytic expulsion should appear more often under those conditions. Moreover, both the aggressive and less aggressive karyotypes of *C. albicans* could be used in co-infection experiments with slow and fast replicating macrophages to simulate the population game. Once the molecular mechanisms of non-lytic expulsion are better understood, overexpression and knockouts could be used to end up in specific Nash equilibria.

Our findings using Game Theory and dynamic optimization give a holistic perspective on fungal infection processes and are consistent with experimental observations [26]. They further explain why the frequency of non-lytic expulsion can be very low in experimental studies. Both our modelling approaches can be used for further studies of other fungal pathogens such as *C. neoformans* and *C. krusei*. For this, our models would need to be adapted to the specific situations of those infections and to the different lifestyles of those pathogens. An extension of our study could be the activation of macrophages through chemokines and cytokines and the recruitment of other immune cells like neutrophils. The question would then be whether it is more beneficial to invest in the attack of fungal cells or in recruitment. Also interesting for further extensions to our model are other macrophage strategies such as macrophage extracellular trap-like structures [11] and the competition within the *C. albicans* population.

## Supplementary Material

Supplementary Material

## Data accessibility

The datasets supporting this article are included in the main paper and/or have been uploaded as part of the electronic supplementary material.
