# Supplementary material for: Modelling the host–pathogen interactions of macrophages and Candida albicans using Game Theory and dynamic optimization
Source: J R Soc Interface. 2017 Jul 12;14(132):20170095. doi: 10.1098/rsif.2017.0095 (PMC5550964; doi:10.1098/rsif.2017.0095)
Supplement: Supplementary Material [file rsif20170095supp1.pdf]

Supplementary Material

Modelling the host-pathogen interactions of macrophages and *Candida albicans* using Game Theory and dynamic optimisation

Sybille Dühring[1], Jan Ewald[1], Sebastian Germerodt[1], Christoph Kaleta[2], Thomas Dandekar[3], and Stefan Schuster[1]

[1]Dept. of Bioinformatics, Friedrich-Schiller-University Jena, Germany
[2]Research Group Medical Systems Biology, Institute for Experimental Medicine, Christian-Albrechts-University Kiel, Germany
[3]Biocenter, Dept. of Bioinformatics and Research Center for Infectious Diseases, Julius-Maximilians-University Würzburg, Germany

For the parameter sensitivity analysis we sampled 1000 parameter sets assuming a log-normal distribution for each parameter, where the respective mode (maximum of the density function) is equivalent to the parameter values in Table 1. Hereafter let τ be the vector containing the parameter values in Table 1:

$$\tau^T = (\alpha, \beta, \gamma, \delta, \epsilon, \mu, \nu, \lambda). \tag{1}$$

To adjust the parameter variance to the magnitude of the parameter value τ_i, the variance of the log-normal distribution is set for each parameter according to:

$$\sigma_i^2 = \sigma_{global}^2 \cdot \tau_i \tag{2}$$

with $\sigma_{global}^2 = 0.1$. For quantification of the parameter sensitivity we calculated the Spearman correlation of the sampled parameter value and the effective replication time $\mathrm{eff}(u(t))$, which combines the amount of replication with the advantage gained compared to a non-replication strategy of macrophages:

$$\mathrm{eff}(u(t)) = (F(1) - F(u(t)) \int_0^T (1 - u(t))dt. \tag{3}$$

This ensures that the correlation is not impaired by optimal strategies involving replication to escape lysis with little effect on the number of *C. albicans* cells as seen in Fig. 5 c in the main text.

We performed the analysis with varying MOIs (1:1 and 3:1) and macrophage replication rates (standard and high).

In the two scatter plots of Fig. S1 the parameter values (y-axis) of each simulation run are plotted against the effective replication times (x-axis) of the corresponding simulation run for a MOI of 1:1. To have a significant correlation the p - value needs to be smaller than 0.05 while an

absolute correlation coefficient rho of greater 0.1 ($rho < |0.1|$) indicates a sensitive parameter. In general, the rho value can take up any number between -1 (perfect negative correlation) and 1 (perfect positive correlation). The colourisation according to the relation of hyphae and macrophages at the end of the simulation shows that for most parameter runs the macrophage population is superior (blue dots). From this we conclude that the macrophage population is capable to control the *C. albicans* population with fungal clearance taking place.

In the two scatter plots of Fig. S2 again the parameter values (y-axis) of each simulation run are plotted against the effective replication times (x-axis) of the corresponding simulation run but considering a MOI of 3:1. This shifts the balance in favour of the fungal population. Compared to Fig. S1 more orange dots are depicted, indicating that the amount of *C. albicans* cells is surpassing the amount of macrophages. Especially for the parameter α and ν we see a phase separation of blue and orange dots. This shows, that the outcome of the simulation run (amount of macrophages compared to the amount of fungal cells) is depending on the parameter value. For high α values there are more blue dots indicating a fungal clearance. For low α values there are more orange dots indicating macrophage death and fungal outgrowth at the end of the simulation run.

In Fig. S3 and Fig. S4 the distribution of the optimal controls $u(t)$ of all simulation runs for each time point are depicted. The black line shows the median of this distribution. Intuitively that is the “middle” value of the data separating the lower and the higher half of the data sample (all controls $u(t)$ of the 1000 simulation runs). The darker grey area indicates where the majority of all controls $u(t)$ of the middle 50 % (that is the difference between the 75 th and 25 th percentiles) of the distributions are. The light grey area indicates the same for the difference between the 90 th and 10 th percentiles.

Fig. S3 a considers a standard replication rate and a MOI of 1:1. The light grey and dark grey areas are exactly in line with the black line of the median. That means that all control variables (ranging from the 10 th to the 90 th percentiles in the distribution) are the same, taking on the value 1. This means that all macrophages are phagocytosing in each time point in our dynamic optimisation model.

Fig. S4 a considers a standard replication rate but this time the balance is shifted in favour of the fungal cells as the MOI is 3:1. Again the dark grey area is exactly in line with the black line of the median indicating that all control variables (ranging from the 25 th to the 75 th percentiles in the distribution) are the same taking on the value 1. There is however a change in the light grey area with $u(t) \sim 0.5$. This means that most of the macrophages are purely phagocytosing in our dynamic optimisation model. However, in 30 % ((25 th percentile - 10 th percentile)+(90 th percentile - 75 percentile)) of the simulation runs we find a optimal control where at least 50 % of the macrophages are phagocytosing.

Since the vast majority of the 1000 parameter sets lead to an optimal strategy without any replication, we increased the reference replication rate to $\alpha = 0.0590$ and repeated the parameter sensitivity analysis for MOI of 1:1 (Fig. S3 b) and 3:1 (Fig. S4 b).

In Fig. S3 b we see that while the median is still unchanged with higher macrophage replication rates the optimal controls $u(t)$ change, so that no longer all macrophages are only phagocytosing but instead some fraction of the macrophage population is undergoing mitosis.

In Fig. S4 b not only the macrophage replication rates are higher but also the balance is shifted in favour of the *C. albicans* population (because of the MOI 3:1). This leads to a drastic change in the optimal control $u(t)$ and therefore in the macrophage behaviour. While for the median roughly 20 % of the macrophages are proliferating in the time between 2 h and 8 h for the 10 - 90 % percentiles, indicated by light grey area, almost 90 % of the macrophages do so.

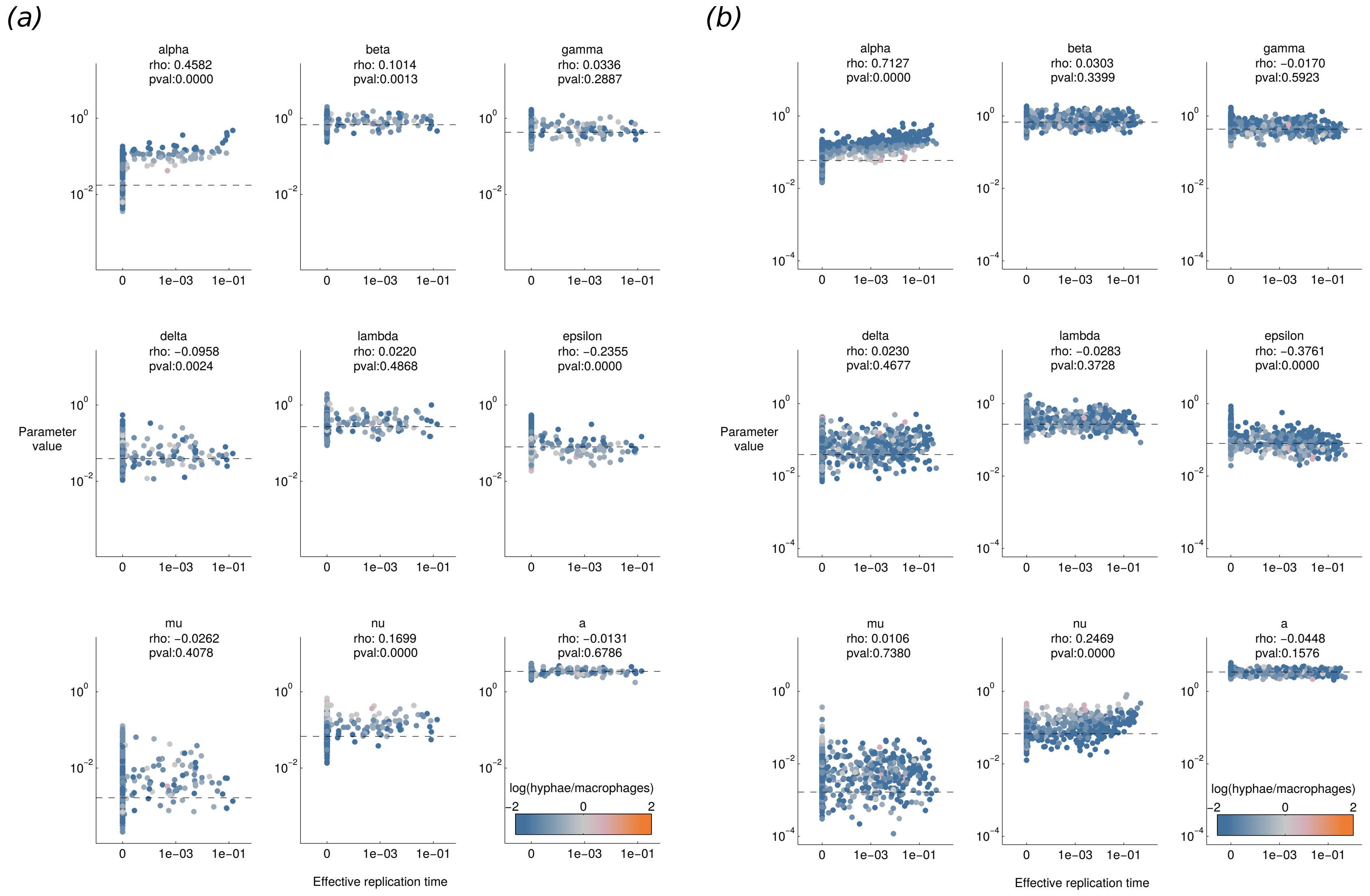

Fig. S1: Parameter sensitivity and the population relations of invasive *C. albicans* hyphae and macrophages at MOI of 1:1 under varying scenarios (standard replication rate *(a)* and high replication rate *(b)*). To visualise the influence of the parameters, the parameter values (y-axis) are plotted against the effective replication times (x-axis). Sensitive parameters are therefore visualised by a positive or negative trend and by the corresponding Spearman correlations depicted in each plot. For nonsensitive parameters dots are horizontally distributed around their reference value (dotted-line). Additionally, the parameter influence on the outcome of simulation is depicted by the hyphae to macrophage ratio at the end of each optimisation run as a coloured point, ranging from orange (superior number of hyphae) to blue (superior number of macrophages).

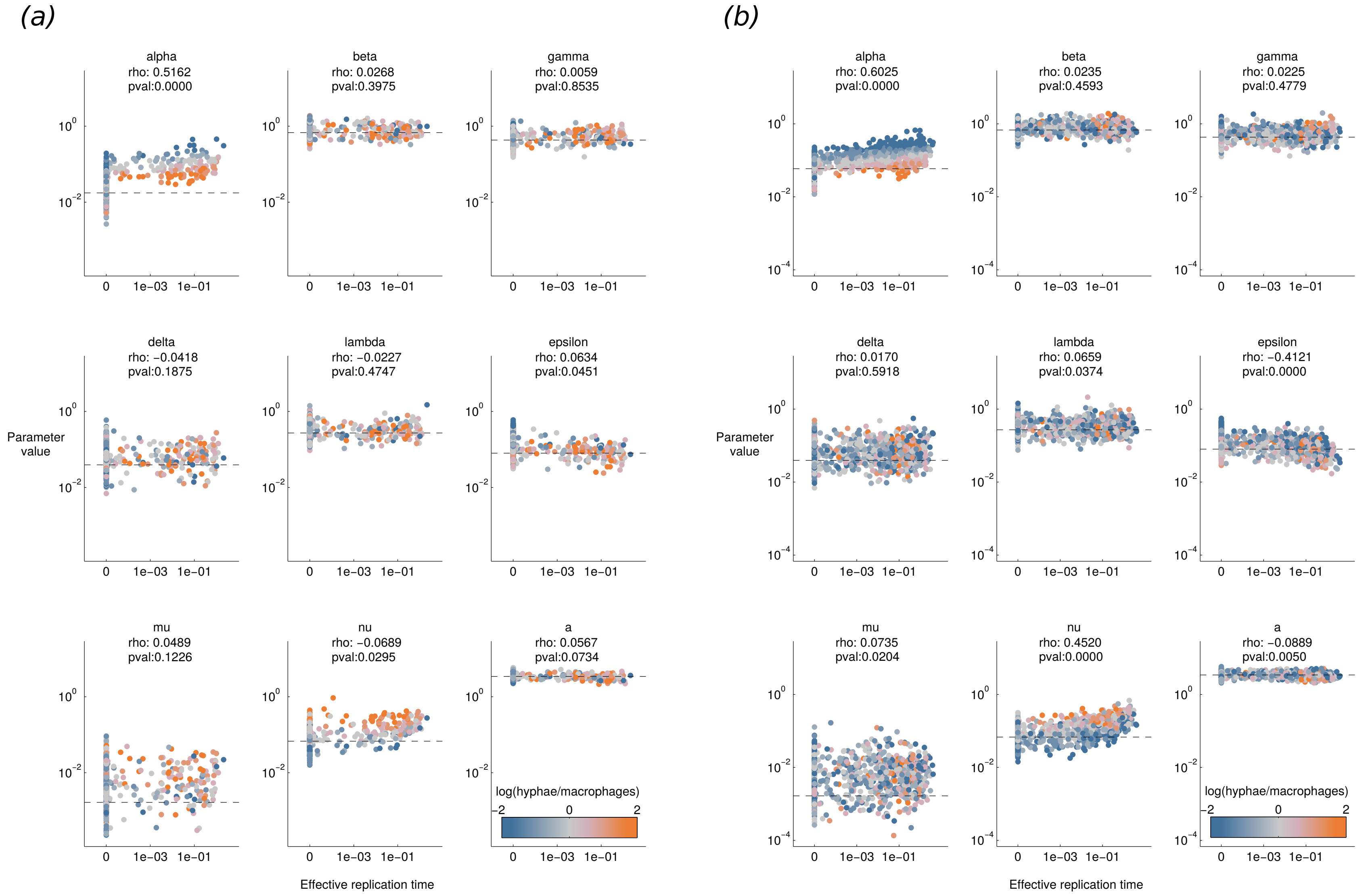

Fig. S2: Parameter sensitivity and the population relations of invasive *C. albicans* hyphae and macrophages at MOI of 3:1 under varying scenarios (standard replication rate *(a)* and high replication rate *(b)*). To visualise the influence of the parameters, the parameter values (y-axis) are plotted against the effective replication times (x-axis). Sensitive parameters are therefore visualised by a positive or negative trend and by the corresponding Spearman correlations depicted in each plot. For nonsensitive parameters dots are horizontally distributed around their reference value (dotted-line). Additionally, the parameter influence on the outcome of simulation is depicted by the hyphae to macrophage ratio at the end of each optimisation run as a coloured point, ranging from orange (superior number of hyphae) to blue (superior number of macrophages).

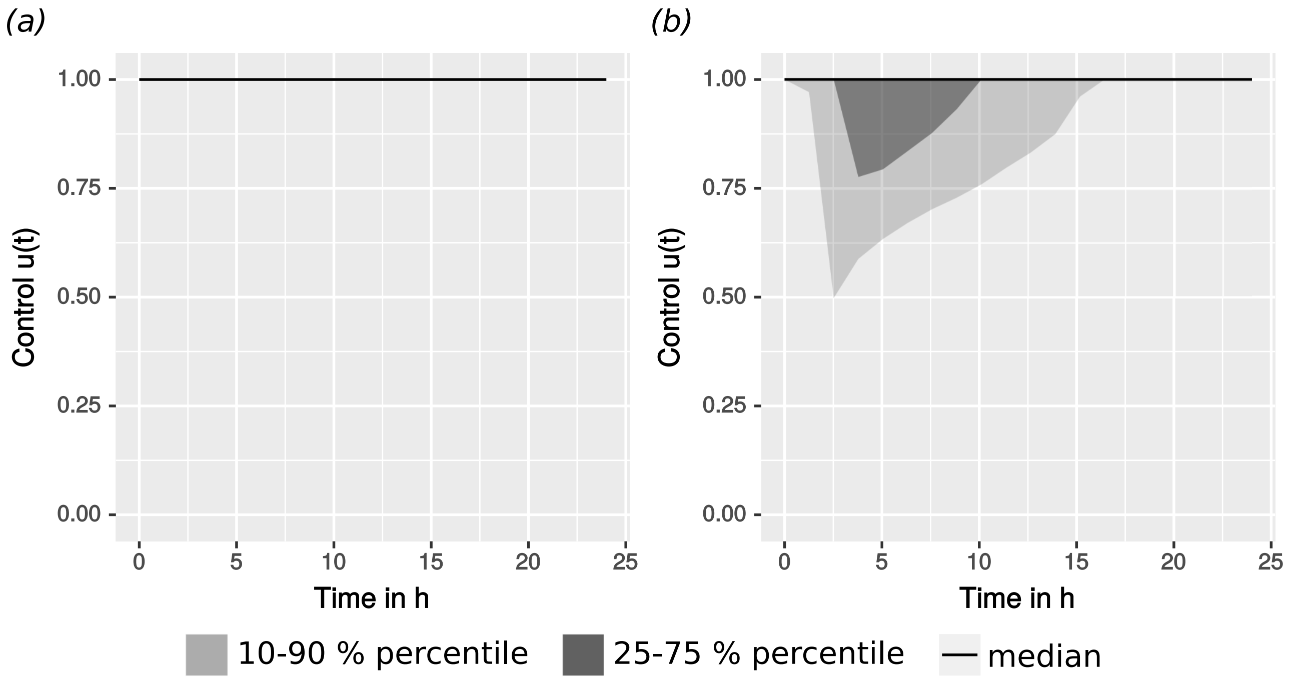

Fig. S3: Distribution of optimal controls $u(t)$ at MOI of 1:1 under varying scenarios (standard replication rate *(a)* and high replication rate *(b)*). Control values of $u(t)$ are the fractions of different macrophage status ranging from 0 (only replication) to 1 (only phagocytosis). The optimal solutions for all parameter runs are used to determine the consensus control and are depicted as shaded grey areas where 80 % (light grey) respectively 50 % (dark grey) of all time courses of controls are captured. The black line represents the median time course of $u(t)$.

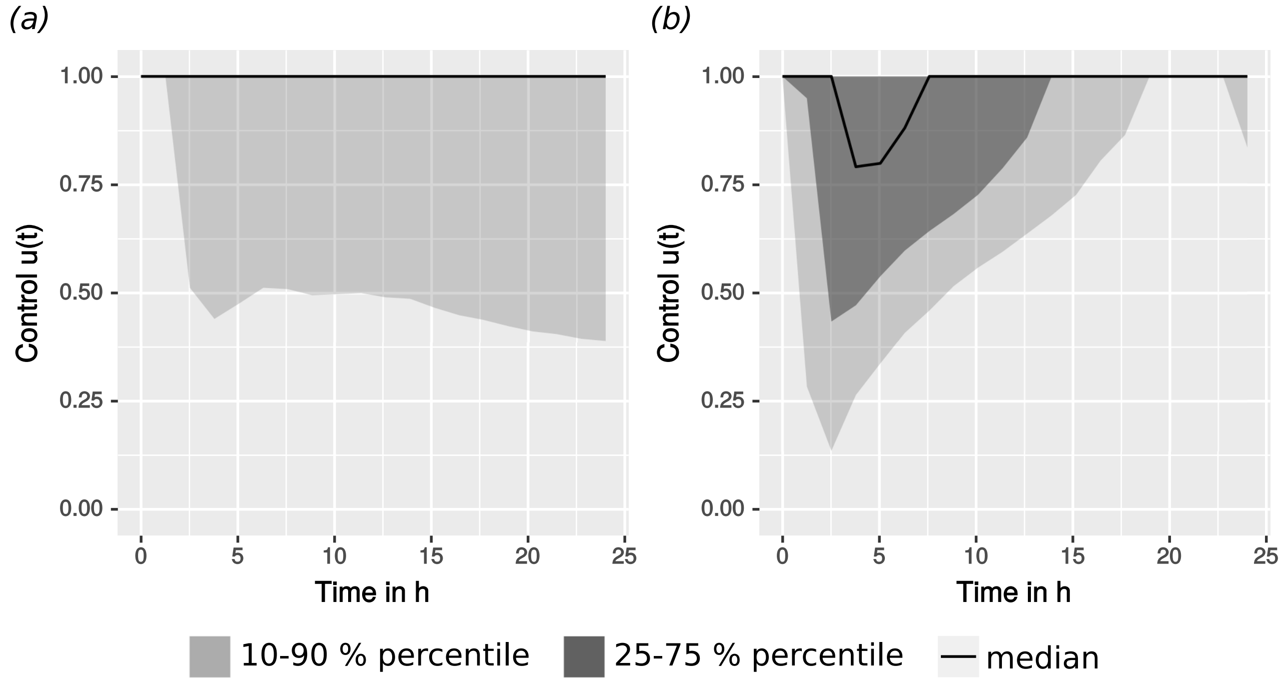

Fig. S4: Distribution of optimal controls $u(t)$ at MOI of 3:1 under varying scenarios (standard replication rate *(a)* and high replication rate *(b)*). Control values of $u(t)$ are the fractions of different macrophage status ranging from 0 (only replication) to 1 (only phagocytosis). The optimal solutions for all parameter runs are used to determine the consensus control and are depicted as shaded grey areas where 80 % (light grey) respectively 50 % (dark grey) of all time courses of controls are captured. The black line represents the median time course of $u(t)$.
